# Supplementary material for: Changes in the Intranetwork and Internetwork Connectivity of the Default Mode Network and Olfactory Network in Patients with COVID-19 and Olfactory Dysfunction
Source: Brain Sci. 2022 Apr 18;12(4):511. doi: 10.3390/brainsci12040511 (PMC9029634; doi:10.3390/brainsci12040511)
Supplement: Supplementary file 1 [file brainsci-12-00511-s001.zip › brainsci-1657846-SI.pdf]

Supplementary materials

Table S1. Anatomic location of the 31 regions of interest used to characterize the olfactory network.

AAL label	Anatomic location	Coordinate			Peak t value
		x	y	z	
3	Left superior frontal gyrus, dorsolateral	-21	42	33	9.3
4	Right superior frontal gyrus, dorsolateral	18	60	12	6.5
5	Left superior frontal gyrus, orbital	-21	21	-12	10.69
6	Right superior frontal gyrus, orbital	18	15	-18	10.44
7	Left middle frontal gyrus	-21	42	30	9.02
8	Right middle frontal gyrus	21	45	27	5.14
9	Left middle frontal gyrus, orbital part	-21	33	-18	8.59
10	Right middle frontal gyrus, orbital part	24	30	-21	8.76
13	Left inferior frontal gyrus, triangular part	-36	27	30	6.78
15	Left inferior frontal gyrus, orbital part	-30	27	-21	10
16	Right inferior frontal gyrus, orbital part	18	18	-18	9.3
21	Left olfactory cortex	-3	15	-3	19.79
23	Left superior frontal gyrus, medial	-15	60	12	8.68
24	Right superior frontal gyrus, medial	15	48	0	8.43
25	Left superior frontal gyrus, medial orbital	-6	42	-12	9
26	Right superior frontal gyrus, medial orbital	15	45	0	9.79
29	Left insula	-24	12	-18	8.61
30	Right insula	24	15	-18	9.3
31	Left anterior cingulate cortex	-6	39	9	8.9
32	Right anterior cingulate cortex	9	36	0	9.79
38	Right hippocampus	21	0	-21	7.31
41	Left amygdala	-18	3	-15	8.93
42	Right amygdala	18	6	-15	10.75

71	Left caudate nucleus	-9	15	0	52.29
72	Right caudate nucleus	9	18	-3	16.55
73	Left putamen	-15	15	0	25.07
74	Right putamen	15	15	-6	13.61
75	Left lenticular nucleus, pallidum	-12	9	0	22.1
76	Right lenticular nucleus, pallidum	12	6	-3	13.5
83	Left temporal pole: superior temporal gyrus	-21	6	-21	9.42
84	Right temporal pole: superior temporal gyrus	21	9	-18	10.67

Table S2. Anatomic location of the 37 regions of interest used to characterize the default mode network

AAL label	Anatomic location	Coordinate			Peak t value
		x	y	z	
3	Left superior frontal gyrus, dorsolateral	-24	36	36	14.5
4	Right superior frontal gyrus, dorsolateral	24	36	39	13.04
7	Left middle frontal gyrus	-24	36	30	14.72
8	Right middle frontal gyrus	24	36	36	13.65
23	Left superior frontal gyrus, medial	0	57	3	15.14
24	Right superior frontal gyrus, medial	3	57	3	15.17
25	Left superior frontal gyrus, medial orbital	0	51	-12	16.78
26	Right superior frontal gyrus, medial orbital	3	51	-12	17.15
31	Left anterior cingulate cortex	-9	48	-3	17.79
33	Left middle cingulate cortex	-12	-51	33	13.22
34	Right middle cingulate cortex	0	-30	33	12.76
35	Left posterior cingulate cortex	-3	-48	21	28.04
36	Right posterior cingulate cortex	3	-42	18	21.89
37	Left hippocampus	-24	-18	-18	7.93
38	Right hippocampus	27	-21	-15	6.66
39	Left parahippocampus	-27	-24	-24	13.77
40	Right parahippocampus	27	-21	-24	12.52
43	Left calcarine fissure and surrounding cortex	-3	-57	12	31.48
44	Right calcarine fissure and surrounding cortex	3	-57	15	38.8
45	Left cuneus	-9	-60	21	23.54
46	Right cuneus	3	-60	21	27.11
47	Left lingual gyrus	-6	-51	3	18.56
48	Right lingual gyrus	6	-54	9	25.96
49	Left superior occipital gyrus	-27	-75	42	6.32
51	Left middle occipital gyrus	-45	-72	27	15.43

52	Right middle occipital gyrus	51	-66	27	9.12
55	Left fusiform gyrus	-27	-27	-24	14.92
56	Right fusiform gyrus	27	-27	-21	11.04
61	Left inferior parietal gyrus	-39	-75	42	11.18
65	Left angular gyrus	-45	-69	27	15.71
66	Right angular gyrus	51	-66	30	9.8
67	Left precuneus	-6	-57	18	55.75
68	Right precuneus	3	-57	18	38.74
82	Right superior temporal gyrus	48	-57	24	10.26
85	Left middle temporal gyrus	-51	-66	21	16.08
86	Right middle temporal gyrus	60	-3	-27	16.35
88	Right temporal pole: middle temporal gyrus	57	9	-33	9.29
